# Aggregation of cohorts for histopathological diagnosis with deep morphological analysis

**DOI:** 10.1038/s41598-021-82642-1

**Published:** 2021-02-03

**Authors:** Jeonghyuk Park, Yul Ri Chung, Seo Taek Kong, Yeong Won Kim, Hyunho Park, Kyungdoc Kim, Dong-Il Kim, Kyu-Hwan Jung

**Affiliations:** 1 VUNO Inc., Seoul, Korea; 2 Pathology Center, Seegene Medical Foundation, Seoul, Korea; 3 Department of Pathology, Green Cross Laboratories, Yong-in, Gyeonggi Korea

**Keywords:** Machine learning, Cancer imaging

## Abstract

There have been substantial efforts in using deep learning (DL) to diagnose cancer from digital images of pathology slides. Existing algorithms typically operate by training deep neural networks either specialized in specific cohorts or an aggregate of all cohorts when there are only a few images available for the target cohort. A trade-off between decreasing the number of models and their cancer detection performance was evident in our experiments with The Cancer Genomic Atlas dataset, with the former approach achieving higher performance at the cost of having to acquire large datasets from the cohort of interest. Constructing annotated datasets for individual cohorts is extremely time-consuming, with the acquisition cost of such datasets growing linearly with the number of cohorts. Another issue associated with developing cohort-specific models is the difficulty of maintenance: all cohort-specific models may need to be adjusted when a new DL algorithm is to be used, where training even a single model may require a non-negligible amount of computation, or when more data is added to some cohorts. In resolving the sub-optimal behavior of a universal cancer detection model trained on an aggregate of cohorts, we investigated how cohorts can be grouped to augment a dataset without increasing the number of models linearly with the number of cohorts. This study introduces several metrics which measure the morphological similarities between cohort pairs and demonstrates how the metrics can be used to control the trade-off between performance and the number of models.

## Introduction

Pathologists diagnose cancer from hematoxylin and eosin (H&E) stained slides after comprehensively analyzing the histological features in a given slide^1^. Likewise, data-driven algorithms including deep learning (DL) have been devised to detect cancer using H&E morphology^2^. While a larger dataset size used to train DL algorithms directly translates to better performance, previous cancer detection models have been trained on cancer-specific datasets such as breast cancer^2–5^, skin cancer^6–8^, lung cancer^9^, bladder cancer^10^, prostate cancer^8,11,12^, stomach cancer^13–15^, colon cancer^14^ and lymph node metastases^8,16,17^, with restricted capacity from limited data. Another approach is to develop a universal model with the hope that increasing the dataset size outweighs the drawbacks brought by introducing irrelevant information or features^18^. However, as will be shown shortly, we witnessed that training a universal model to detect cancer from H&E images collected from various cohorts results in worse performance than the average performance achieved by cancer-specific models. This prompts the following question: when and how can datasets be aggregated to better detect cancer from fewer dataset configurations?

At first sight, it may be tempting to conclude that training models on cohorts with similar histologies would enhance their performance. However, dataset aggregation based on subjective evaluation of morphological similarity can result in inclusion of irrelevant information that outweighs the benefits brought by diversity. It would also be desirable if cohorts could be grouped objectively by similarity without pathologists’ expertise. In this study, we show how cohort aggregation can be done appropriately without any domain knowledge by introducing several metrics inspired by domain adaptation^19^ (see “Materials and methods” section), and that the performance of DNNs can be improved with a reduced number of models.

**Figure 1:**
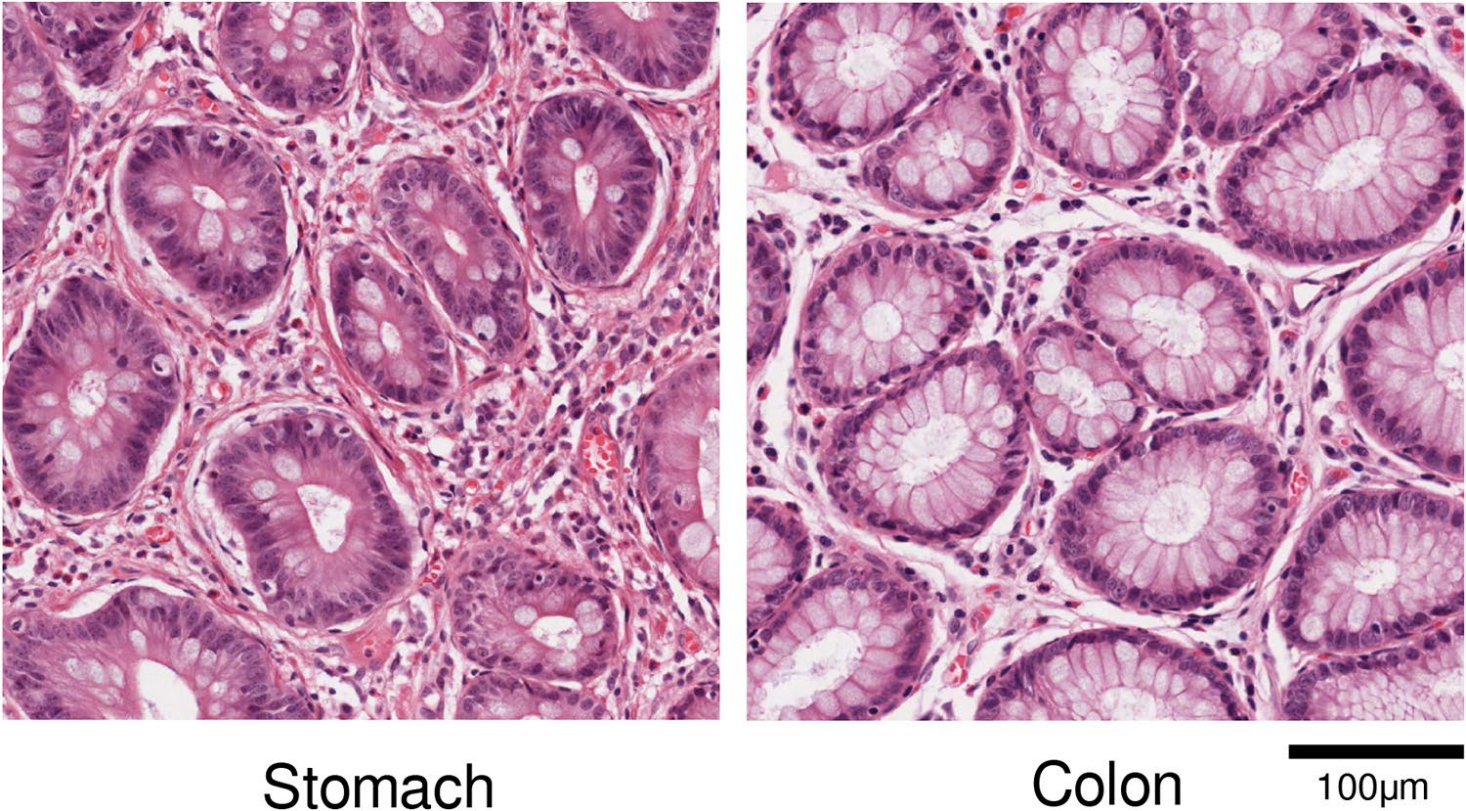
Examples of H&E slide patch from gastric and colonic tissues. On the left are gastric glands with intestinal metaplasia, and on the right are colonic glands. The two tissues share similar histological features.

We explored the effect of combining datasets belonging to different cohorts in developing a cancer diagnostic model from H&E images. Our study was conducted using The Cancer Genome Atlas (TCGA) dataset comprised of 37 cohorts across 33 cancer types^20^. This dataset has been used in various studies for stain color handling^21^, microsatellite instability prediction^22^, tissue classification^23^ and gene mutation prediction^9^. We included 12 TCGA cohorts which contain at least 36 normal slides: (KIRC, Kidney renal clear cell carcinoma; LIHC, Liver hepatocellular carcinoma; THCA, Thyroid carcinoma; OV, Ovarian serous cystadenocarcinoma; LUAD, Lung adenocarcinoma; LUSC, Lung squamous cell carcinoma; BLCA, bladder urothelial carcinoma; UCEC, Uterine corpus endometrial darcinoma; BRCA, Breast invasive carcinoma; PRAD, Prostate adenocarcinoma; COAD, Colon adenocarcinoma; STAD, Stomach adenocarcinoma) as summarized in Table 1. Note that some cohorts in this dataset such as COAD and STAD are known to share similar morphologies as illustrated in Fig. 1. After validating the diagnostic performances of cohort-specific models and comparing their performance with that of a universal model trained on all 12 cohorts, we show how a careful aggregation guided by our metrics can be used to reduce the number of models and computational cost for training while retaining the high performance achieved by the specialized models. In one aspect, our work is an extension of that conducted by Kather et al.^22^ who trained a microsatellite instability (MSI) detection model on gastric formalin-fixed paraffin-embedded (FFPE) slides and validated the model on colorectal FFPE slides, demonstrating the applicability of DL-based classification on morphologically similar cohorts. In contrast to their experiments, we show that a direct usage of both source and target cohorts guided by carefully designed metrics can be used to automate the cohort aggregation process.

**Table 1:** The Cancer Genome Atlas (TCGA) cohorts and the number of frozen slide images used in the study. The slides with sample type codes 01 (primary solid tumor) and 11 (solid tissue normal) are referred to as positive and negative slides, respectively.

Cohort	Number of positive slides	Number of negative slides
KIRC	1089	564
LIHC	399	89
THCA	534	97
OV	1175	163
LUAD	819	244
LUSC	753	347
BLCA	431	37
UCEC	750	54
BRCA	1572	399
PRAD	604	118
COAD	871	109
STAD	632	123

Cohort abbreviations are as follows: *KIRC* kidney renal clear cell carcinoma, *LIHC* liver hepatocellular carcinoma, *THCA* thyroid carcinoma, *OV* ovarian serous cystadenocarcinoma, *LUAD* lung adenocarcinoma, *LUSC* lung squamous cell carcinoma, *BLCA* bladder urothelial carcinoma, *UCEC* uterine corpus endometrial carcinoma, *BRCA* breast invasive carcinoma, *PRAD* prostate adenocarcinoma, *COAD* colon adenocarcinoma, *STAD* stomach adenocarcinoma.

## Results

**Figure 2:**
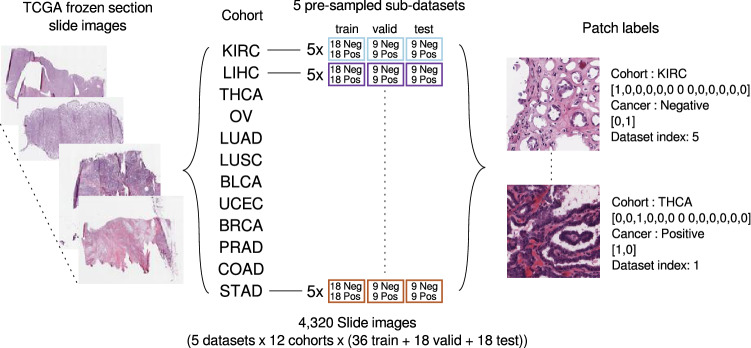
Dataset preparation: a total of 4,320 frozen slide images were used for train/validation/test. Five sub-datasets were constructed for each cohort, with each sub-dataset composed of randomly sampled slides numbering (cancer detection and general cohort discrimination) 18 positive and negative slides or (positive/negative cohort discrimination) 36 positive or negative slides, with replacement between sub-datasets. Each patch was annotated indicating its cohort, presence of cancer, and sub-dataset index.

**Figure 3:**
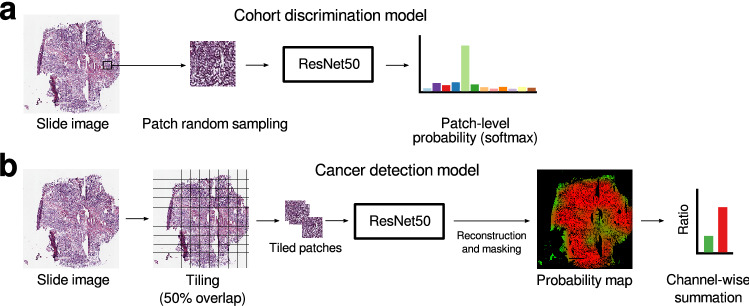
Input/output schematic of deep learning models. Cohort discrimination model (**a**) predicts which cohort a patch is drawn from. Cancer detection model (**b**) makes slide-level predictions determining whether the slide contains cancer or not.

We conducted several experiments using the dataset and model setups (Figs. 2 and 3) to test if prior histology knowledge of morphological similarities among different cancer types could be used to train DL models efficiently. Our first experiment demonstrates the sub-optimality of using a single universal model trained on all H&E images regardless of their cohorts of origin. Three DL models were then trained to discriminate a slide’s cohort of origin based on positive (cancer present), negative (cancer absent), or either type of slides, where intuitively, they would have difficulty distinguishing cohorts with similar morphological structures. This idea stems from the ${\mathscr{H}}$-divergence introduced in the context of domain adaptation^19^, where the difficulty of discriminating between two domains (i.e. cohorts) can be used to understand the feasibility of training on a source cohort for the task on a target cohort. The models obtained from these experiments were then used to guide the aggregation of cohorts. Our aggregation method allows controlling the trade-off between performance and number of models, with models trained on cohort groups generally achieving higher performance as the number of models increases (i.e. with higher specialization).

### Specialized and universal cancer detection models and morphological similarities among cohorts

**Figure 4:**
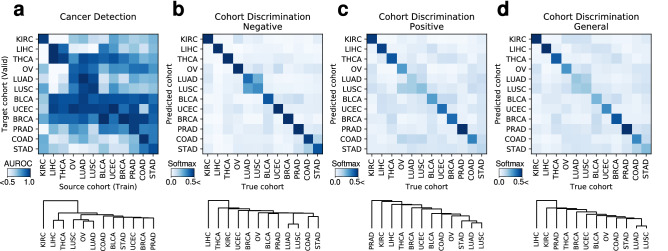
Similarity matrices induced by (**a**) single cohort cancer detection and (**b**–**d**) cohort discrimination models (top) and their corresponding hierarchical clustering analysis trees (bottom). Single-cohort cancer detection models *C* (**a**) with columns indicating source (train) cohorts and rows indicating target (validation) cohorts. Negative cohort discrimination model $D_n$ (**b**), positive $D_{p}$ (**c**) and general $D_{g}$ (**d**) with columns indicating cohort of origin and rows indicating the model’s predictions.

To compare the performances of cohort-specific and universal cancer detection models, we first trained 12 cohort-specific models to diagnose cancer from H&E images in designated cohorts. A universal model was then trained on an aggregate of all 12 cohorts, and its area under the receiver operating characteristic (AUROC) scores were obtained when tested on each of the 12 cohorts. Indeed the average AUROC of cohort-specific models (the average AUROC 0.9687 ± 0.0173, ±:95% confidence interval) far exceeds that of the universal model (the average AUROC 0.8570 ± 0.0702), demonstrating how merging cohorts with distinct morphologies yields highly sub-optimal performance. Furthermore, cross-cohort validation was performed using the 12 cohort-specific cancer detection models to obtain a $12 \times 12$ similarity matrix (Fig. 4a) and an ordered tree ${\mathscr{T}}_{c}$ obtained via hierarchical clustering analysis (HCA). Cohorts, or leaf nodes, at greater depths in the tree share a common morphological structure with neighboring cohorts, whereas shallow leaf nodes are unique. Furthermore, the distance (number of intermediate vertices in the shortest path) between the cohorts in the tree indicate their morphological dissimilarity.

The diagonal entries in the similarity matrix show that cohort-specific models generally perform best when the target cohort matches the source cohort as expected. The low AUROCs in off-diagonal entries show the degree of incompetence of cohort-specific models when tested on other cohorts. Interestingly, most models performed well on BLCA, UCEC, and BRCA cohorts, indicating how these three contain simple cancer morphology that is relatively easy to infer. Cohort groupings ${\mathscr{T}}^{C}$ obtained from HCA show that KIRC is unique, standing out from the other cohorts of interest. On the other hand, ovarian epithelial cancers and pulmonary adenocarcinomas can display similar histological features of adenocarcinoma, which may have contributed to the high similarity between OV and LUAD in the similarity matrix.

### Cohort discrimination models and morphological similarities among cohorts

Next, we measured the morphological similarities among cohorts using cohort discrimination models. Three similarity matrices and HCA trees ${\mathscr{T}}^{D_{n}}, {\mathscr{T}}^{D_{p}}, {\mathscr{T}}^{D_{g}}$ were obtained as cohort-discrimination performances of models $D_{n}$, $D_{p}$, $D_{g}$ trained to classify the cohort of origin based on negative, positive, or either (general) slides, respectively. Before presenting our main results, we describe the intuition connecting the cohort discrimination task in determining which cohorts are similar, which in turn is used to aggregate cohorts and control the trade-off between performance and number of models. Pathologists agree that some organs (e.g. stomach and colon, Fig. 1) share similar histological structures that may not be easily distinguished to the untrained eye. If this is an inherent difficulty that cannot be overcome by any expressive model, DNNs would also struggle in discriminating such images, and adding the extra images from another cohort would be similar to simply increasing the number of training samples in the original cohort of interest without any expense of adding irrelevant information. The cohort discrimination performances thus measure the morphological similarity between cohort pairs, with a pair being similar when positive, negative, or general slides from the cohorts are hard to differentiate.

**Figure 5:**
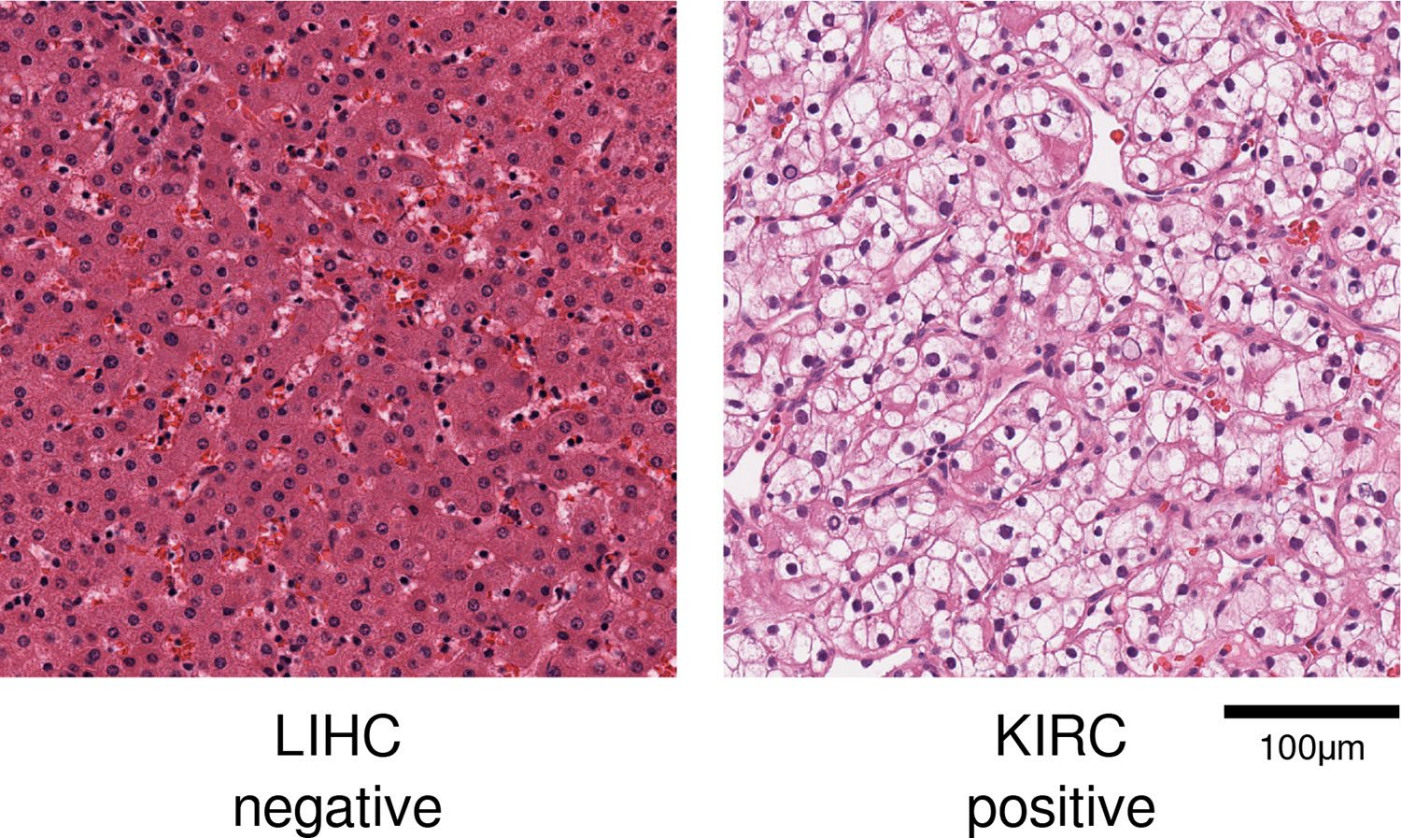
Representative images of the morphological features unique to LIHC negative and KIRC positive slides are shown. LIHC (negative) shows cords of reddish polygonal hepatocytes, and KIRC (positive) shows clear cell clusters with distinct cellular borders.

These cohort discrimination models’ one-versus-all classification performances are displayed in Fig. 4b–d along with their HCA groupings. We had expected cohorts from the same organ (e.g. lung cohorts LUSC and LUAD) to be adjacent in the negative tree ${\mathscr{T}}^{D_{n}}$, as negative H&E images do not have cancer-related patterns useful for differentiating the organ of origin. As expected, COAD–STAD and LUSC–LUAD which are biologically similar or originate from the same organs, were paired when cancer was absent (${\mathscr{T}}^{D_{n}}$, Fig. 4b), showing that gastrointestinal tract cohorts and the two lung cohorts were similar in morphology. The latter pair was also grouped nearly by ${\mathscr{T}}^{C}, {\mathscr{T}}^{D_{p}}$ (Fig. 4a,c), implying their morphological similarity even when cancer was present. This observation is in line with clinical knowledge that pulmonary adenocarcinomas can be difficult to distinguish from pulmonary squamous cell carcinomas when it histologically shows poor differentiation. The liver and kidney renal clear cell carcinoma have unique tissue structures (Fig. 5), and we had expected the cohort discrimination models to distinguish these cohorts with high accuracy, and the resulting HCA grouping to isolate these cohorts. LIHC and KIRC were isolated (have depth 1 and 2) in ${\mathscr{T}}^{D_{n}}$ and ${\mathscr{T}}^{D_{p}}$, indicating H&E images from LIHC and KIRC with and without cancer, respectively, have substantially different morphologies from other cohorts. Meanwhile, THCA (depth 2 in ${\mathscr{T}}^{D_{n}}$) and PRAD (depth 1 in ${\mathscr{T}}^{D_{p}}$) were also isolated, and it may be due to the unique follicular structure in THCA and the uniformly small glandular structure in PRAD that may have led to such result.

### Qualitative analysis of morphological similarity among cohorts

**Figure 6:**
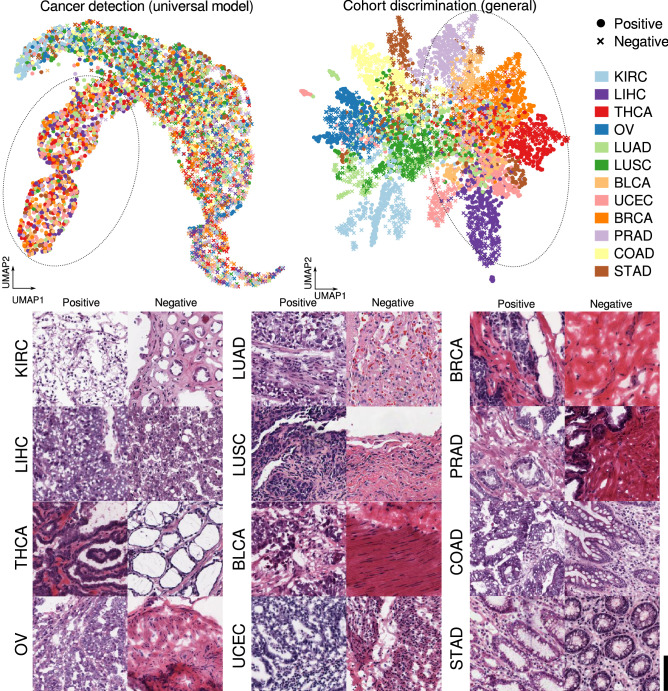
Uniform manifold approximation and projection (UMAP) visualizations for features extracted by the universal cancer detection model and general cohort discrimination model. Identical patches were used for both visualizations. Dotted ellipses indicate cohort mixtures (THCA–BLCA–UCEC–BRCA–PRAD–LIHC). Sampled positive and negative patches of all cohorts are shown below. KIRC is composed of clear cells and LIHC of eosinophilic polygonal cells that are distinct from others. THCA, OV, UCEC, and PRAD all show an adenocarcinoma pattern with differences in glandular contour and cytologic features. BLCA shows solid cancer cell nests. BRCA can manifest as both solid cell nests as in this patch or an adenocarcinoma pattern composed of small glands. STAD and COAD show relatively well-differentiated tall tubular structures. Scale bar: 100 $\upmu$m.

To qualitatively analyze the similarities among cohorts, we visualized the features extracted by the universal model and general cohort discrimination model using Uniform Manifold Approximation and Projection (UMAP) (Fig. 6; Materials and methods)^24^ Both UMAP visualizations projected features from STAD and COAD close to each other; however, there was still a clear distinction between the two. This demonstrates how STAD and COAD are similar in morphology, but have unique traits not shared between the two. THCA and BRCA are on the other end of the spectrum, with the pair being distinguished from others, but their features overlapping substantially. Both the universal cancer detection and general cohort discrimination models isolated KIRC, indicating that KIRC shares the least amount of morphological properties with other cohorts. Surprisingly, while LIHC has a unique hepatic tissue structure absent in all other cohorts considered, it is only isolated when features were extracted using the cohort discrimination model and is rather uniformly distributed when extracted by the universal model. In general, cohort mixtures (THCA–BLCA–UCEC–BRCA–PRAD–LIHC) formed by the universal model were similar to those obtained from the cohort discrimination model explicitly trained to differentiate cohorts, even though the universal model had no prior knowledge that the aforementioned cohorts have related or unrelated morphology. This suggests the similarity/dissimilarity among cohorts via independent dimension reduction techniques.

### Aggregating cohorts while retaining performance

**Figure 7:**
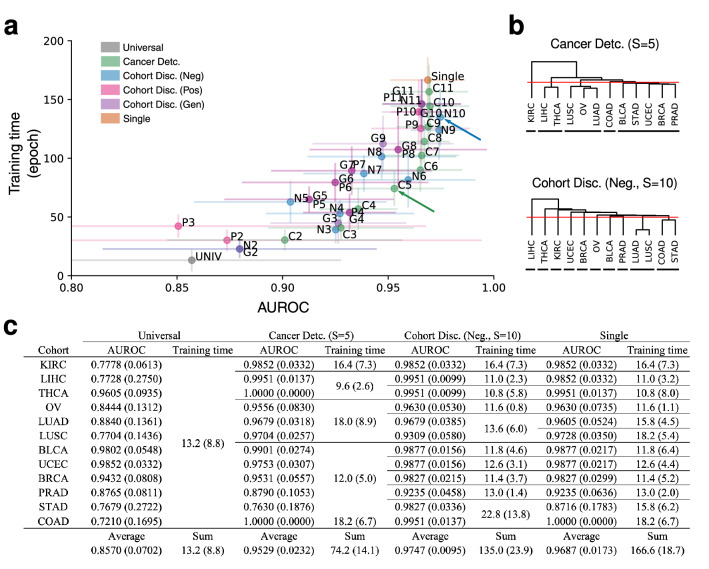
(**a**) AUROC scores and training time of universal model, single models, and super-cohort models on the test set of each cohort. Lines represent 95% confidence intervals. Superscripts indicate the models referenced to aggregate cohorts, and *S* is the number of super-cohorts. C, ${\mathscr{T}}^{C}$; N, ${\mathscr{T}}^{D_{n}}$, P, ${\mathscr{T}}^{D_{p}}$; G, ${\mathscr{T}}^{D_{g}}$ (e.g., C5:${\mathscr{T}}^{C} (S=5)$). (**b**) Super-cohort configuration of the super-cohort ${\mathscr{T}}^{C} (S=5)$ incurring least training time among those that performed better than the 95% lower CI of cohort-specific models (top) and super cohort ${\mathscr{T}}^{D_{n}} (S=10)$ which resulted in best performance (bottom). Hierarchy level (red lines) and cohorts included in super cohorts (black lines) are shown. (**c**) Comparison of naive combinations (universal model and single models) and super-cohort models indicated by arrows in (**a**). See Supplementary Data Table s2 for details of all super-cohort models. *Detc.* detection, *Disc.* discrimination, *Pos* positive, *Neg* negative, *Gen* general.

Using the four aforementioned models and their corresponding measures of cohort similarity, we trained and evaluated the performance of DNNs trained on groups of cohorts (hereafter referred as super-cohorts) obtained from the four respective groupings. As shown in Fig. 7a, the average AUROC of DNNs generally increased as the models specialized in specific cohorts. Among the models that performed within 95% confidence interval (CI) of single cohort models’ AUROC, we report the per-cohort performances of (1) super-cohort models that resulted in minimum training time (${\mathscr{T}}^{C} (S=5)$, where S is the number of super-cohorts) and (2) best performing super-cohort models (${\mathscr{T}}^{D_{n}} (S=10)$). Training time $t({\mathscr{T}}) := \sum _{s \in {\mathscr{T}}} {\bar{t}}(f_s)$ refers to the sum of super-cohort models’ training times $t(f_s)$ averaged over 5 trials (sub-datasets). The models of $S=5$ super-cohorts ${\mathscr{T}}^{C}$ showed average AUROCs of 0.9529 ± 0.0232, and sum of average training time was 74.2 epochs reducing 55% of training time compared to the specialized models (Fig. 7c). Furthermore, we looked whether super-cohort models could outperform the specialized models. The models of $S=10$ super-cohorts ${\mathscr{T}}^{D_{n}}$ showed AUROCs of 0.9747 ± 0.0095 which outperformed the specialized models. This demonstrates the need to handle dataset configurations more carefully when applying DL to cancer detection, especially to avoid drawing conclusions based on sub-optimal models. The list of all of the super-cohorts and test results are shown in Supplementary Data Table s2.

## Discussion

Constructing datasets composed of multiple cohorts with similar morphologies can be useful for the following reasons. First, utilization of a vast amount of publicly available data from cohort of similarity can increase a DNN’s performance trained using DL algorithms. Second, appropriate aggregating of cohorts can reduce the number of diagnostic models required to diagnose a given histologic slide image: a physican does not need to synchronize the algorithms across cohorts whenever new data is added to the training set. Lastly, it can be used to develop an algorithm for rare cancers for which there does not exist a large number of histologic images, using datasets from more well-known cancer types that bear histologic resemblance.

Previous DL algorithms in digital pathology developed models without considering morphological similarities or dissimilarities among cohorts^2–17^. While several studies already demonstrated the morphological similarities using cross-cohort classification^22,25^ and pan-cancer/tissue classification^18,23^, our work takes an additional step to investigate how information extracted from such experiments can be used to improve classification-efficiency tradeoff, by fixing the performances as a distance metric and training on super-cohort groupings obtained from HCA. Each cross-validation performance can be seen as a single entry in the cancer-detection confusion matrix in our work, and we believe our experiments subsume previous methods as one component of analysis, rather than as comparative baselines. To the best of our knowledge, this is the first study to assess cohort aggregation with systematical analysis of cohort morphologies by cancer type using modern data-driven methods. We trained DNNs on 4 different tasks to express the similarities among cohorts as features extracted from the trained models, which in turn was used to produce hierarchical clusters. The obtained clusters were used to effectively group cohorts such that models trained on these groupings perform as well as or better than both the universal model and the most specialized, single-cohort models (Fig. 7c).

We address some possible extensions for future work. Our study considered aggregating datasets to improve the performance and computational cost of cancer detection model. A natural extension of the current study would be to see if the proposed metrics still prove useful in detecting cancer using other cohorts of interest. Another course for future work would be applying our framework to other datasets. Concurrent with our work, Hosseini et al.^26^ published an annotated pathological image database with hierarchical ordering and it would be interesting to see if the cohorts’ properties remain unchanged when our framework is applied to their dataset. Recall that similarity among cancer tissues’ features affects the cancer detection models more than normal organ features: the grouped cohort models’ performances showed a higher correlation with the positive discrimination model’s metric than the negative model’s. This suggests that in order to develop accurate cancer detection models, it is crucial to allocate distinct models for different cancer types regardless of which organ the tissues were collected from. Our dataset combination may be used to develop models that not only classifies cancer types accurately but also provides genetic information as in the study by Fu et al.^18^ who merged 28 TCGA cohorts to train a model for tissue/cancer classification that also provides genetic and survival information.

Transfer learning is often used to improve performance when the task of interest lacks data. In an extended experiment, we observed that a ImageNet pre-trained universal model achieved an average AUROC of $0.9414\,\pm \,0.0220$ for a total time of $9.8\,\pm \,1.8$ epochs. This is a considerable improvement over the universal model’s performance trained from random initialization, but doesn’t quite reach the performance of specialized (super-cohort or single) models. Moreover, transfer learning is orthogonal to the cohort aggregation scheme suggested in this work, and may be applied to super-cohort and single models to further increase the overall performances in Fig. 7.

The current study has a few limitations. All patches were labeled corresponding to the slide labels resulting in noisy targets for supervised learning. This may have been problematic because some patches used to train the positive cohort discrimination model $D_{p}$ and cancer detection models *C* may not have contained cancer despite the fact that they were labeled otherwise. We may have obtained different results had we used a weakly supervised algorithm or if we had acquired precise annotations by pathologists. The quality of patches was inconsistent; if we had sampled patches acquired from identical medical centers, with more slides available for training/testing to account for the deficient number of training/testing samples, we may have reached different conclusions.

In conclusion, we showed that deep learning algorithms can provide objective measures of morphological similarity among multiple cohorts, and that based on this similarity among cohorts, aggregating cohorts can be done successfully for more efficient development of high-performance diagnostic deep learning algorithms.

## Materials and methods

### Dataset

TCGA slide images were used to train and test our cancer detection models. Despite the higher quality of formalin-fixed paraffin-embedded (FFPE) images, we used only the frozen tissue images since the TCGA dataset contains an insufficient number of negative FFPE images. Among the cohorts present in the TCGA dataset, cohorts with less than 36 positive (cancer present, sample type code 01: primary solid tumor) and 36 negative (cancer absent, code 11: solid tissue normal) slides were removed, leaving a total of 12 cohorts: KIRC, LIHC, THCA, OV, LUAD, LUSC, BLCA, UCEC, BRCA, PRAD, COAD, STAD (Table 1).

To balance the number of positive and negative samples for each cohort in training the cancer detection models, we randomly sampled 5 sub-datasets composed of 36 training (18 positive, 18 negative) and 36 held-out (18 positive, 18 negative) slides, with each sub-dataset satisfying all of the criteria below:

- Train and held-out sets do not overlap at the patient level.
- For each positive or negative slide, at most one slide was sampled from a given patient for a maximum of two slides per patient.
- The held-out set was randomly divided into validation and test sets with equal classes.

More details can be found in the Supplementary Data Table s1 with the slide names and sub-dataset partitions. The cohort discrimination models were also trained using a similar dataset partition: 18 and 36 slides per cohort for the positive/negative discriminative and general (positive and negative) models, respectively, and half for each validation and test sets. All experiments were conducted independently on the 5 sub-datasets.

### Training and inference details

All models shared the same ResNet-50 V2 architecture operating on patches evenly cropped from slides with spatial resolution $\Omega = \{1, \ldots , 224\}^2$, where each pixel spans 1.2 $\upmu$m, and were trained to make slide-level predictions for both cohort discrimination and cancer detection tasks^27^. For training, each patch’s label was assigned its corresponding slide-level ground-truth (i.e. cohort type for the cohort discrimination task and cancer presence/absence for cancer detection). Upon inference, the slide-level prediction ${\hat{y}} \in \{0,1\}$ was computed using a likelihood-ratio test ${\hat{y}} = {\mathbf {1}}\left\{ {\hat{p}}_{1} / {\hat{p}}_{0} \ge \eta \right\}$ for some threshold $\eta \ge 0$ that determines the operating point on the ROC curve, and the model’s pixel-wise predictions $[p_{y} (i,j)]_{(i,j) \in \Omega }$ were summed channel-wise to obtain the class confidences ${\hat{p}}_{y} \,{\overset{\Delta }{=}}\, \sum _{i,j} p_{y} (i,j), \forall y \in \{0,1\}$.

All models were trained using the Adam optimizer (learning rate $10^{-3}$) from identical, randomly initialized up to equivalent architectures (i.e. output dimension) until the validation accuracy saturated for 5 epochs with batch size 32. One epoch is defined as 1000 training iterations (32,000 patches), and the models were validated on 6400 randomly sampled patches at the end of each epoch. Data augmentation was performed using the following: all input patches were rotated by multiples of 90 degrees and also on the horizontally flipped. Augmentation was performed following Liu et al.^17^ in the following order: maximum brightness change of 64/255, saturation $\le \, 0.25$, hue $\le \, 0.04$, contrast $\le \, 0.75$, and the resulting pixels were clipped to values in [0, 1]. Our implementation was based on Tensorflow^28^.

### Cohort discrimination models and domain adaptation/generalization

The cohort discrimination tasks resemble the ${\mathscr{H}}$-divergence $d_{\mathscr{H}}$ introduced for a remote task known as domain adaptation and generalization^19^. In particular, this metric in its original context quantifies the disparity between two domains (in our context, cohorts) characterized by their distributions $P_{i}$ and $P_{j}$ over possible images. Borrowing from its original context, Ben–David showed that a small ${\mathscr{H}}$-divergence between two cohorts characterized by $P_{i}$ and $P_{j}$ signifies that a model trained on an aggregate of cohort i and j will likely achieve small error when tested on either cohort. An exact computation of ${\mathscr{H}}$-divergence requiring infinite validation samples is impossible, but its estimate ${\hat{d}}_{\mathscr{H}}(P_{i},P_{j})$ computed over a finite sample size can be used instead. The general discrimination model $D_{g}$ directly estimates the ${\mathscr{H}}$-divergence in a pairwise manner, i.e. if instances from cohort $P_{i}$ are often (mis-)classified as coming from $P_{j}$, cohort i is similar to cohort j. In contrast, the negative and positive discrimination models $D_{n}, D_{p}$ are conditional variants of ${\mathscr{H}}$-divergence, conditioned on the fact that the input image is either negative or positive . The confusion matrices corresponding to $D_{n}$, $D_{p}$, and $D_{g}$ were constructed using 54,000 patches (100 patches/slide, 9 slides, 12 cohorts, 5 sub-datasets) for the first two and 108,000 patches (18 slides) for $D_{g}$.

### Aggregating cohorts

Hierarchical clustering analysis (HCA) was performed using Ward’s method with the Euclidean distance between column vectors corresponding to cohorts. Each element $v_{ij}$ of the $j{\text{th}}$ column vector $v_{j}$ was quantified as one of the following (1) cancer detection performance on cohort *i* when the model was trained on cohort *j*, (2) confidence of a cohort discrimination model, trained to differentiate cohorts solely on a positive slide’s morphology, that positive slides from cohort *i* were sampled from cohort *j*, (3) negative cohort discrimination model’s confidence similar to (2) but tested on negative slides, and (4) general cohort discrimination model’s confidence across both positive and negative slides. All performances were obtained on the validation set for the purpose of aggregating cohorts. The resulting dendrogram was cut into non-overlapping super-cohort groups, aggregating morphologically similar cohorts and excluding dissimilar cohorts (see Fig. 7b). Performances reported in Fig. 7 are $S=5$ and $S=10$ super-cohort cancer detection models obtained using Alg. 2 trained with a number of super-cohorts *S* on ${|}N{|}$ cohorts with reference models *C* (cancer detection) and $D_{n}$ (negative cohort discrimination) models, respectively. 
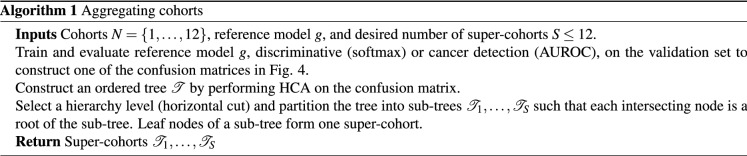

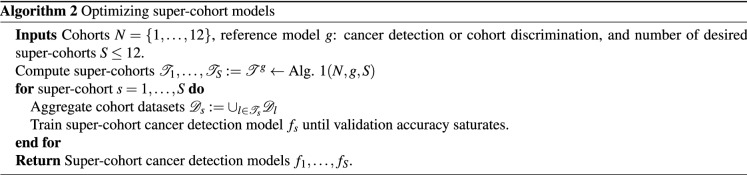




### Visualization details

The uniform manifold approximation and projection (UMAP) visualization was attained using custom parameters (number of neighbors $= 20$, minimum distance $= 0.5$) on the features extracted from the penultimate layer of the universal model for training set of sub-dataset 1. Interpreting the visualization is difficult when using excessive number of slides, and we instead randomly sample 20 patches per slide from the training sub-dataset to extract the features for this visualization.

## Supplementary information

Supplementary Information 1.

Supplementary Information 2.

Supplementary Information 3.

## Data Availability

All TCGA slide images are publicly available on the GDC Data Portal (https://portal.gdc.cancer.gov).
