# Supplementary material for: Aggregation of cohorts for histopathological diagnosis with deep morphological analysis
Source: Sci Rep. 2021 Feb 3;11:2876. doi: 10.1038/s41598-021-82642-1 (PMC7858624; doi:10.1038/s41598-021-82642-1)
Supplement: Supplementary file 1 — Supplementary Information 1. [file 41598_2021_82642_MOESM1_ESM.docx]

**SUPPLEMENTARY MATERIALS**

**Title:** Aggregation of cohorts for histopathological diagnosis with deep morphological analysis

**Authors:** Jeonghyuk Park^1,+,^*; Yul Ri Chung^2,+^, Seo Taek Kong^1^, Yeong Won Kim^1^, Hyunho Park^1^, Kyungdoc Kim^1^, Dong-Il Kim^3^, Kyu-Hwan Jung^1^

**Author affiliations:** ^1^VUNO Inc., Seoul, Korea, ^2^Pathology Center, Seegene Medical Foundation, Seoul, Korea, ^3^Department of Pathology, Green Cross Laboratories, Yongin, Gyeonggi, Korea,

^+^co-first author

*jh.park@vuno.co

**Supplementary Data Table S1**. Details of dataset configuration. Names of the TCGA slides are presented with sub-dataset number, set (train/validation/test), class (positive/negative), and cohort name. The slide level and slide level mpp (micron per pixel) indicate slide level used for patch extraction and resizing.

**Supplementary Data Table S2**. Performances and training times of all super-cohort models. Model names are abbreviated in accordance with Figure 7. Cohort names in a bracket indicate the cohorts included in a super-cohort.
